# Floquet-engineered quantum walks

**DOI:** 10.1038/s41598-020-74418-w

**Published:** 2020-10-16

**Authors:** Haruna Katayama, Noriyuki Hatakenaka, Toshiyuki Fujii

**Affiliations:** 1 Graduate School of Integraged Arts and Sciences, Hiroshima University, Higashihiroshima, 739-8521 Japan; 2 Department of Physics, Asahikawa Medical University, Midorigaoka-higashi, Asahikawa 078-8510 Japan

**Keywords:** Physics, Quantum physics, Quantum simulation, Theoretical physics

## Abstract

The quantum walk is the quantum-mechanical analogue of the classical random walk, which offers an advanced tool for both simulating highly complex quantum systems and building quantum algorithms in a wide range of research areas. One prominent application is in computational models capable of performing any quantum computation, in which precisely controlled state transfer is required. It is, however, generally difficult to control the behavior of quantum walks due to stochastic processes. Here we unveil the walking mechanism based on its particle-wave duality and then present tailoring quantum walks using the walking mechanism (Floquet oscillations) under designed time-dependent coins, to manipulate the desired state on demand, as in universal quantum computation primitives. Our results open the path towards control of quantum walks.

## Introduction

The random walk^1^ is a fundamental concept that has been used to describe a variety of systems with inevitable stochastic processes^2,3^. A classical walker moves one step to the left *or* one step to the right depending on the outcome of a coin toss. After many coin tosses, the walker’s position is random, but is most likely to be close to the start point. In contrast, a walker in a quantum world^4,5^ simultaneously travels in both directions, behaving as a quantum wave, forming a coherent superposition of left and right that occupies more than one location at any given time. In addition, quantum interference effects between the possible trajectories of the walker also contribute to modify the resulting dynamics substantially such as delocalization over many steps. Thus, the wave nature of the walker in *the particle-wave duality* is important for its walking behavior.

The unique features emerging from the wave nature of the quantum walk (e.g., fast spreading^6,7^) have been predominantly applied to quantum search algorithms^8–10^ in quantum information sciences. Recently, quantum walks have been shown to be universal computational primitives in quantum computation, i.e., any quantum algorithm can be reconstructed as a quantum walk algorithm^11,12^. Therefore, precise control of quantum state transfer between arbitrary distant sites is critical for quantum information processing^13–16^. It is, however, not easy to manipulate quantum states using quantum walks due to their essentially random nature^17–19^. In addition, the walking mechanism in the wave picture is less obvious than that in the particle picture, where the walker shifts its position depending on the coin.

One possible approach to find the walking mechanism in the wave picture for controlling quantum walks might be to manipulate the coin transformation which would then allow us to drive walk evolution in a desired manner. Initially, a space-dependent coin was introduced in the quantum walk^20^. Later, a quantum walk with *time*-dependent coins was studied^21–25^. The resulting probability distribution for the quantum walk changed significantly. This implies that coin transformation is certainly involved in the walking mechanism and explicitly designing the sequence of coin transformations could lead to a desired state transfer.

## Results

### The time-dependent coined quantum walk

Suppose a walker on a line with a coin living in the Hilbert space of the whole system ${{\mathscr {H}}}={{\mathscr {H}}}_p\otimes {{\mathscr {H}}}_c$, where ${{\mathscr {H}}}_p$ and ${{\mathscr {H}}}_c$ are the Hilbert space of walker’s position with basic vectors $\{|{m}\rangle ,m\in {\mathbb {Z}}\}$ and of the coin with basic vectors $\{|{L}\rangle ,|{R}\rangle \}$, respectively. The behavior of the walker in the quantum-mechanical domain is described by two operators. One is the shift operator ${\hat{S}}$ defined as

1 $$\begin{aligned} {\hat{S}}=\sum _m\left[ |{L}\rangle \langle {L}|\otimes |{m-1}\rangle \langle {m}|+|{R}\rangle \langle {R}| \otimes |{m+1}\rangle \langle {m}|\right] \end{aligned}$$

which shifts walker’s position. Another is the coin operator which transforms the internal state of the coin. Here, we employ the time-dependent coin,

2 $$\begin{aligned} {\hat{C}}= \begin{pmatrix} \sqrt{\rho (t)}&{} \sqrt{1-\rho (t)} \\ \sqrt{1-\rho (t)}&{} -\sqrt{\rho (t)} \\ \end{pmatrix}, \end{aligned}$$

where $0\le \rho (t)\le 1$. In each step, the walker shifts based on the new internal state of the coin after the transformation from the previous state of the coin. Therefore, the state of the whole system after *n* steps starting from the initial state $|{\psi }\rangle _0$ is given by

3 $$\begin{aligned} |{\psi }\rangle _n=[{\hat{S}}({\hat{C}}\otimes {\mathbb {I}})]^n|{\psi }\rangle _0 = \sum _{m=-n}^{n}[L_{m,n}|{m,L}\rangle +R_{m,n}|{m,R}\rangle ], \end{aligned}$$

where the probability amplitudes $L_{m,n}$ and $R_{m,n}$ are given as

4 $$\begin{aligned} R_{m,n+1}&=\sqrt{\rho (nT)}R_{m-1,n}+\sqrt{1-\rho (nT)}L_{m-1,n}, \end{aligned}$$

5 $$\begin{aligned} L_{m,n+1}&=\sqrt{1-\rho (nT)}R_{m+1,n}-\sqrt{\rho (nT)}L_{m+1,n}. \end{aligned}$$

Here ${\mathbb {I}}$ is the unit matrix and *T* is the time between steps. Finally, the probability distribution of finding the walker in the position *m* after *n* steps, $P_m(n)$, is given as $$P_m(n)=P_m^L(n)+P_m^R(n) \ $$ with $P_m^L(n)=|L_{m,n}|^2$ and $P_m^R(n)=|R_{m,n}|^2$.

### The numerical simulation of the time-dependent coined quantum walk

**Figure 1:**
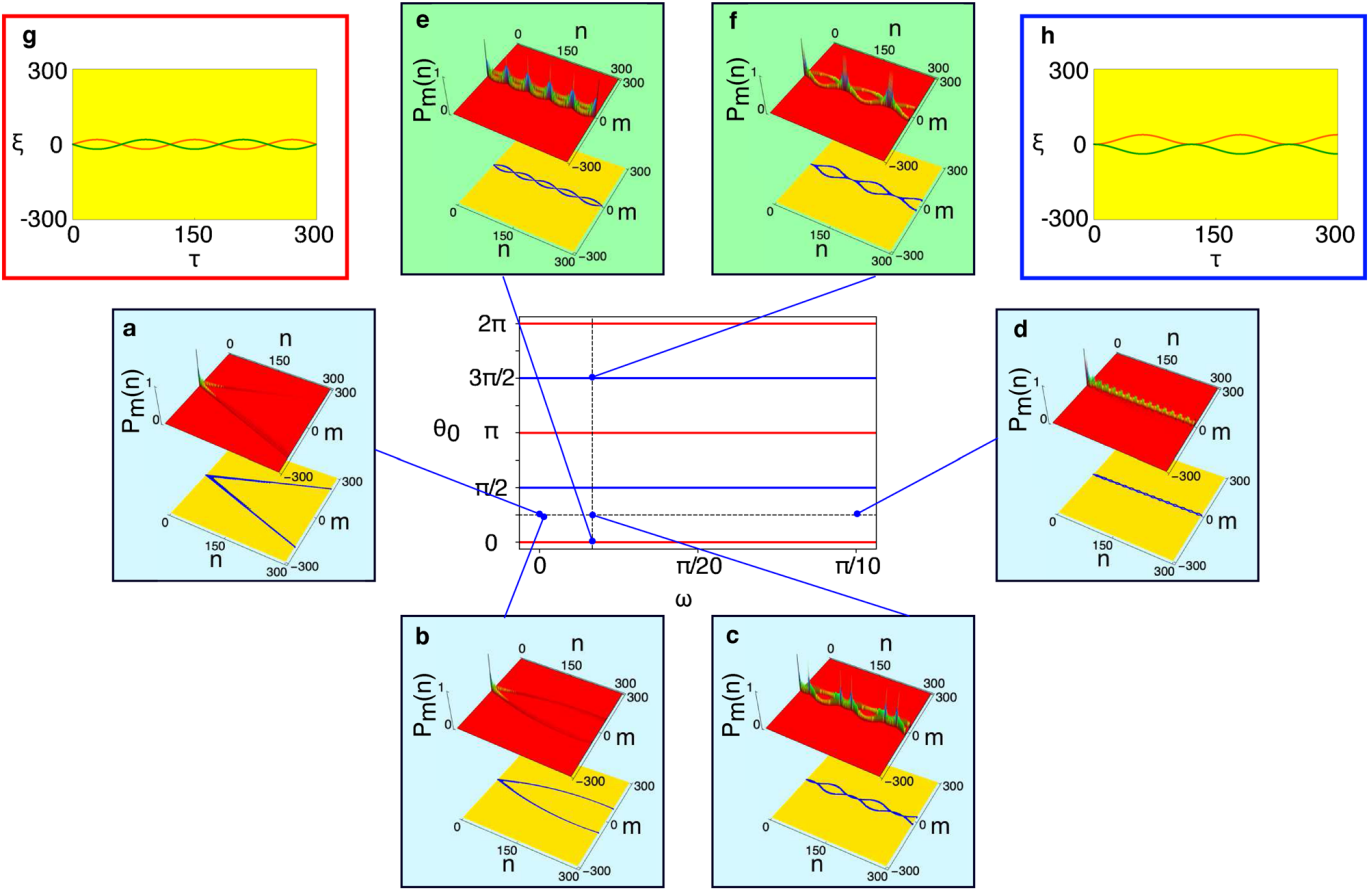
A wide variety of the walker’s trajectories. The phase diagram (center) with insets indicating probability distributions of walker’s position (upper) and trajectory (lower) over 300 steps at typical parameters for coin with initial phase $\theta _0$ and frequency $\omega$, by numerical simulations starting with the initial state of $|{\psi }\rangle _0=|{0,R}\rangle /\sqrt{2}+i|{0,L}\rangle /\sqrt{2}$. (**a**) linear spreading $\theta _0=\pi /4$ and $\omega =0$, (**b**) curved spreading $\theta _0=\pi /4$ and $\omega =\pi /1000$, (**c**) a loop-line chain $\theta _0=\pi /4$ and $\omega =\pi /60$, (**d**) a localized line $\theta _0=\pi /4$ and $\omega =\pi /10$, (**e**) a crossing loop-loop chain $\theta _0=0$ and $\omega =\pi /60$, (**f**) a touching loop-loop chain $\theta _0=3\pi /2$ and $\omega =\pi /60$, (**g**) walker’s trajectories in a crossing loop-loop chain predicted by the analytic solution, (**h**) walker’s trajectories in a touching loop-loop chain predicted by the analytic solution. Loop-loop chains appear as crossing chains *only* at $\theta _0=n\pi$ (red lines) and as touching chains *only* at $\theta _0=(2n+1)\pi /2$ (blue lines) in the phase diagram (where $n=$integer).

Applying the above definitions, we performed numerical simulations on the quantum walk with the time-dependent coin. We employ $\rho (t)=\cos ^2(\theta _0+\omega t)$ as an example of the time-dependent coin characterized by the coin flipping frequencies $\omega$ and the initial phases $\theta _0$. We discovered numerically for the first time that the probability distributions of finding a particle (walker) on a line as a function of both $\omega$ and $\theta _0$ parameters exhibit a wide variety of *walker’s trajectories* representing high probability portions such as loops connected by lines (referred to as loop-line chains, see Fig. 1c) and adjacent loops (loop-loop chains, Fig. 1e,f) as shown in Fig. 1.

With the time-independent coins $(\omega =0)$, as shown in Fig. 1a, a trajectory with linear spreading in space peculiar to the quantum walk is reproduced, supporting our numerical simulations. With the time-dependent coins with finite frequencies $(\omega \ne 0)$, the trajectory forms closed loops. Interestingly, the resulting closed trajectories are not just sequential loops, as expected naturally, but also occur as connected structures of loops alternating with lines (loop-line chains). In addition, loop size tends to decrease as the coin’s frequency increases. Eventually, a linear trajectory with almost no spatial spread can be observed. Moreover, trajectories are also influenced by the initial phase $\theta _0$. The trajectories in Fig. 1c,e,f with different initial phase values differ from each other even though they have the same $\omega$ value. Note that the trajectories in Fig. 1e,f are categorized as loop-loop chains, but the origin of the loops is completely different as discussed later.

**Figure 2:**
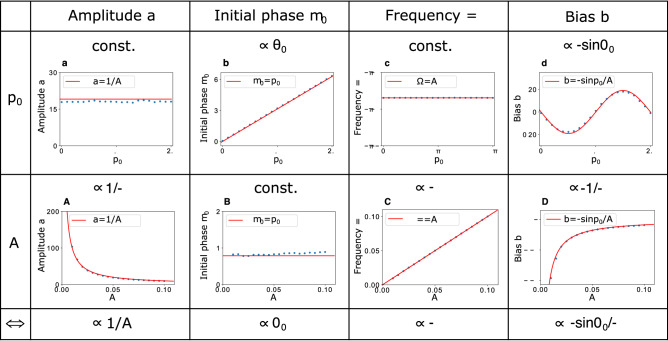
Fitting parameters. The fitting parameters of a trajectory in the form of $x_c=a\sin {(\phi _0+\Omega t)}+b$ are represented by blue points (the numerical simulation results) and a red line (the analytical result) in each graph. The parameters (the amplitude *a*, initial phase $\phi _0$, frequency $\Omega$, and bias *b*) are shown as a function of both the initial phase $\theta _0$ with fixed frequency $\omega =\pi /60$ (top row) and the frequency $\omega$ with an initial phase of $\theta _0=\pi /4$ (bottom row). (**a**) and (**A**) for the amplitude, (**b**) and (**B**) for the initial phase, (**c**) and (**C**) for the frequency, and (**d**) and (**D**) for the bias.

It was found that these features can be reproduced with a simple formula in a numerical analysis, i.e., the trajectory $x_c$ is well fitted by the sinusoidal function expressed as $x_c=a\sin (\phi _0+\Omega t)+b$ where *a*, *b*, $\phi _0$ and $\Omega$ are the amplitude, the bias, the initial phase and the frequency, respectively. Figure 2 shows the fitting parameters *a*, $\phi _0$, $\Omega$, and *b* as a function of both the initial phase $\theta _0$ with fixed frequency $\omega =\pi /60$ (top row) and the frequency $\omega$ with an initial phase of $\theta _0=\pi /4$ (middle row). From each pair of the $\theta _0$ and $\omega$ rows, the physical origin of the fitting parameters can be inferred as shown in the bottom row. These fitting parameters provide an excellent clue to elucidating the walking mechanism.

### The analytic solution of the time-dependent coined quantum walk

Here we derive the analytic solution to a quantum walk with a time-dependent coin based on the seminal work by Knight et al.^26^ together with its extended work to time-dependent coins by Banũls et al.^21^, in order to explore the walking mechanism of its trajectories. From the time evolution of the probability amplitudes in Eqs. (4) and (5) by setting $\rho (nT)=\cos ^2\theta _n$ with $\theta _n=\theta _0+n\omega T$, we obtain the difference equation,

6 $$\begin{aligned} A_{m,n+1}-A_{m,n-1}&=-\cos {\theta _n}(A_{m+1,n}-A_{m-1,n}), \end{aligned}$$

where $A=L, R$. By introducing a continuum field of space and time $A^{\pm }(x,t)$ to take the wave propagation in both positive and negative directions into account^26^, we obtain the differential equation within the long-wavelength approximation,

7 $$\begin{aligned} \frac{\partial }{\partial \tau }A^{\pm }(\xi ,\tau )=\mp \cos {\theta (\tau )} \left( \frac{\partial }{\partial \xi }+\frac{1}{3!}\frac{\partial ^{3}}{\partial \xi ^{3}}\right) A^{\pm }(\xi ,\tau ), \end{aligned}$$

where $\xi$ and $\tau$ are normalized coordinate and time, respectively. The analytic solution is expressed by

8 $$\begin{aligned} A^{\pm }(\xi ,\tau )&=\frac{1}{2}\sum _{m}(A_{m,0}\pm A_{m,1})Z^{\pm }(\xi -m,\tau ), \end{aligned}$$

with

9 $$\begin{aligned} Z^{\pm }(\xi ,\tau )&=\left| \frac{2}{s(\tau )}\right| ^{\frac{1}{3}}e^{\chi }{{\text {Ai}}}(\zeta ), \end{aligned}$$

10 $$\begin{aligned} \zeta&=\left| \frac{2}{s(\tau )}\right| ^{\frac{1}{3}}\left( \pm \xi - s(\tau ) +\frac{2w^4}{s(\tau )}\right) ,\end{aligned}$$

11 $$\begin{aligned} \chi&=\frac{2 w^2}{s(\tau )}\left( \pm \xi - s(\tau )+\frac{4 w^4}{3s(\tau )} \right) ,\end{aligned}$$

12 $$\begin{aligned} s(\tau )&=\mp \int _{0}^{\tau }\cos {\theta (\tau ')}d\tau ', \end{aligned}$$

where ${{\text {Ai}}}(x)$ stands for the Airy function and 2*w* is the standard deviation of the Gaussian function introduced to describe spreading waves. A detailed solution to Eq. (7) is provided in the Method. The probability distribution $P(\xi ,\tau )$ is then obtained by $P(\xi ,\tau )=P^{L}(\xi ,\tau )+P^{R}(\xi ,\tau )$ where $P^{A}(\xi ,\tau )=|A^{+}(\xi ,\tau )+(-1)^{n}A^{-}(\xi ,\tau )|^2$ with $A=L, R$. These results are essentially the same as Bañuls’s except for the difference in the coin operators.

**Figure 3:**
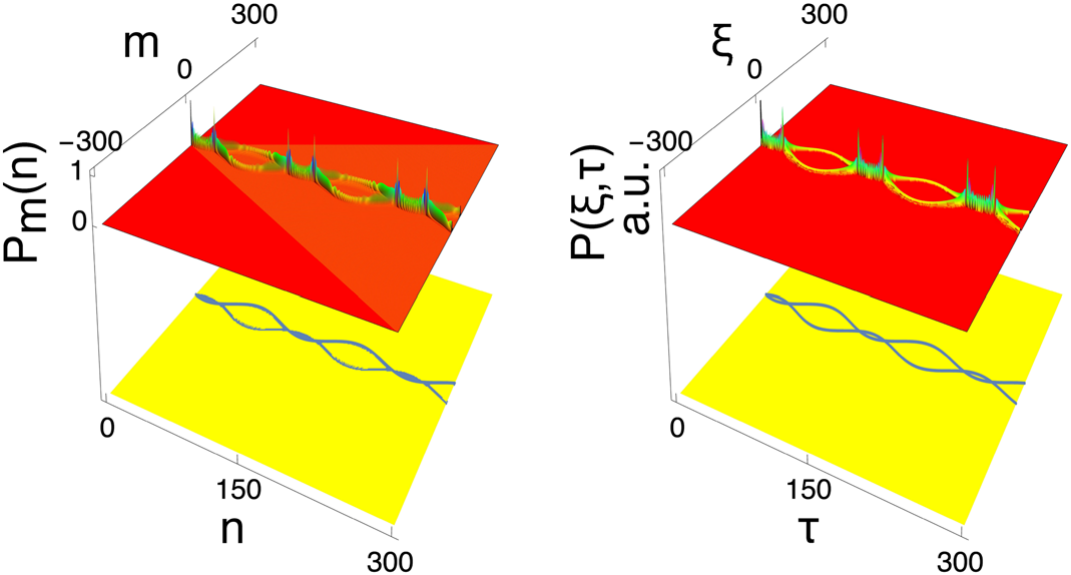
The numerical simulation and the corresponding analytical solution. Probability distributions and their trajectories for the quantum walk with initial state $|{\psi }\rangle _0=|{0,R}\rangle /\sqrt{2}+i|{0,L}\rangle /\sqrt{2}$ ($R_{0,0}=1/\sqrt{2}$ and $L_{0,0}=i/\sqrt{2}$), coin initial phase $\theta _0=\pi /4$, and frequency $\omega =\pi /60$ over 300 steps. The graph on the left shows the result of the numerical simulation, and the graph on the right shows the result of the analytic solution with $w=0.4$. Note that the linear spatial spreading is shown in the probability distribution of the numerical simulation only.

Figure 3 shows the probability distributions of finding the walker on a line, based on the numerical simulation and the analytical solution. It can be seen that the analytical solutions agree well with the numerical solutions, showing that the long-wavelength approximation analysis works very well. However, there is one missing link between the two solutions. In the analytical solution, the linear spreading inherent in the quantum walk is not reproduced and remains unsolved.

### Walking mechanism

Now let us discuss walking mechanism that has not been clarified so far by considering the walker’s trajectories. The essentials of the probability distribution are described by $A^{\pm }(\xi ,\tau )$ expressed in the Fourier form as

13 $$\begin{aligned} A^{\pm }(\xi ,\tau )&=\int _{-\infty }^{\infty }e^{ik\xi }A^{\pm }(k,\tau )dk =B^{\pm }\left( \xi \mp \int _{0}^{\tau }\cos {\theta (\tau ')}d\tau ',\tau \right) , \end{aligned}$$

by using the shifted probability amplitude $B^{\pm }(\xi ,\tau )$

14 $$\begin{aligned} B^{\pm }\left( \xi ,\tau \right) =\int _{-\infty }^{\infty }e^{ik\xi } \left( e^{\pm \frac{1}{6}ik^3\int _{0}^{\tau } \cos {\theta (\tau ')}d\tau '}A^{\pm }(k,0)\right) dk. \end{aligned}$$

Equation (13) means that the entire distribution $A^{\pm }\left( \xi ,\tau \right)$ shifts with $\mp \int _{0}^{\tau }\cos {\theta (\tau ')}d\tau '$ over time. Therefore the trajectory is given by a simple sinusoidal function

15 $$\begin{aligned} x_c&=\mp \int _{0}^{\tau }\cos {\theta (\tau ')}d\tau ' =\mp \left( \frac{1}{\omega }\sin {(\theta _0+\omega \tau )}-\frac{1}{\omega }\sin {\theta _0}\right) . \end{aligned}$$

This analytical result shows complete agreement with the results obtained by numerical analysis.

This agreement allows us to interpret the various trajectories seen in Fig. 1 using the analytical solution. Surprisingly, all the trajectories can be described by two sinusoidal functions in Eq. (15). The bias $b=-\sin {\theta _0}/\omega$ determines the relative overlap between two sinusoidal functions, leading to various types of chain structures. The crossing loop-loop chains with loops of the same size as shown in Fig. 1g appear when the overlaps occur at $\theta _0=n\pi$ $(n=\text{ integer})$ depicted by the red lines in the middle panel of Fig. 1. In addition, at $\theta _0=(n+1)\pi /2$ (the blue lines in the middle panel of Fig. 1), a second type of chain is created by adjacent rather than intersecting sinusoidal functions as shown in Fig. 1h, otherwise the loop-line chains appear instead. Actually, the linear part that seems to be a straight line is the area where the sinusoidal functions overlap. Therefore, the loop-line structure can be described as a chain of loops of two sizes that alternate. On the other hand, frequency $\omega$ determines the size of the loop that depends on $\omega$ in time and $1/\omega$ in space. Thus, the loop becomes smaller when the frequency increases, resulting in narrower trajectories. In this way, through the analytical solution, we obtain a unified view of the probability distribution trajectory shown in Fig. 1.

Now let us discuss the physical origin of trajectories obtained above. Based on Eq. (7), the quantum walk eventually behaves like a quantum-mechanical wave. According to wave theory, there is a well-known dispersion relation, $\omega = v k$, between the wave number *k* and frequency $\omega$. From Eq. (7), the component of the coin operator, $\mp \cos \theta (\tau )$, appears in the place corresponding to the velocity of this wave. This shows that the coin in the wave picture of the quantum walk plays the role of the speed of the quantum wave that determines the walking trajectory. This is the walking mechanism of the quantum walk hidden behind the wave picture. This is one of our central results of this paper.

This correspondence can also be confirmed in the particle picuture. We reconsider the walking mechanism based on the Hamiltonian formalism from the viewpoint of the particle nature in the particle-wave duality. The Hamiltonian for generating one-dimensional discrete time quantum walk can be derived from the walker’s time evolution operator $W\equiv SC=\exp [-iH\tau ]$ for each step of the walk on the discrete position space and is given as $H(k)={\varvec{h}}(k)\cdot {\varvec{\sigma }}$ in the two-component Dirac-like Hamiltonian form where ${\varvec{h}}(k)$ and ${\varvec{\sigma }}$ are a three-dimensional wavenumber *k*-dependent vector and the vector of the Pauli spin matrices, respectively. Specific expressions for the coin operators we have adopted are given in the reference^27^ creating cat states in one-dimensional quantum walks using delocalized initial states.

Since the vector ${\varvec{h}}(k)$ contains information on the coin operator depending on the periodic function in time, the Hamiltonian naturally has the translational invariant under a discrete time translation $H(\tau )=H(\tau +T)$. This allow us to use the Floquet formalism. According to the Floquet theorem, the Floquet state solution $\Phi _\alpha (x,t)$ for the quasienergy $\epsilon _\alpha$ is given by the solution of the eigenvalue equation ${{{{\mathscr {H}}}}_F}(x,t)\Phi _\alpha (x,t)=\epsilon _\alpha \Phi _\alpha (x,t)$ with the Floquet Hamiltonian ${{{{\mathscr {H}}}}_F}=H(x,t)-i\partial /\partial t$.

For our system, the quasienergy $\epsilon _{\pm }$ for the 1*st* Floquet zone^27^ is given in the same approximation as the long wavelength approximation adopted above:

16 $$\begin{aligned} \epsilon _{\pm }=\mp \left( k\cos \theta +\frac{\pi }{2} \right) +O(k^3). \end{aligned}$$

Since the velocity operator is given by the Heisenberg equation ${\hat{v}}=\dot{{\hat{x}}}=[{{{{\mathscr {H}}}}_F},{\hat{x}}]$, the diagonal terms of the velocity representing the left and right propagating waves read $\langle {\hat{v}}_{\alpha }\rangle = v_{\alpha }=d\epsilon _\alpha /dk$. From these, we reconfirm that the velocity of the particle coincides with the wave velocity $\mp \cos \theta$ obtained in the wave picture. Therefore, quantum walk with time-dependent coins can be regarded as a quantum particle oscillating in the Floquet band. This is the counterpart of quantum walks with *space-dependent* coins causing Bloch oscillations in periodic band structures.

### The general time-dependent coined quantum walk

**Figure 4:**
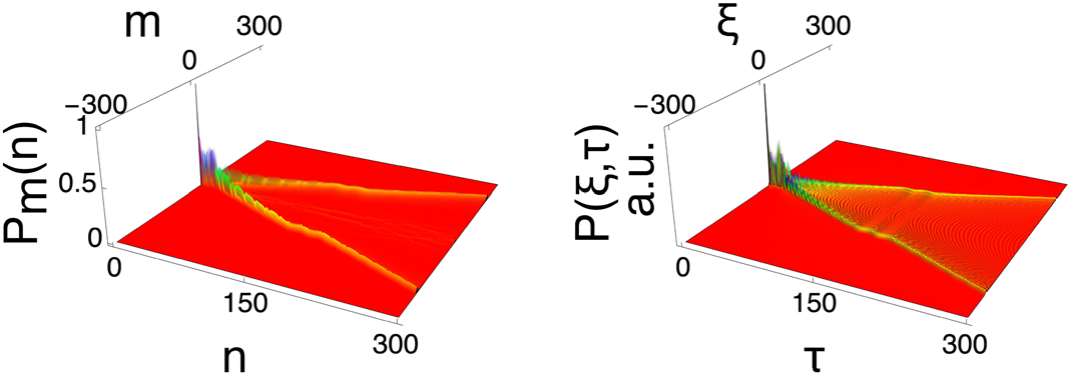
Probability distributions of quantum walks with a general time-dependent coin. Probability distributions of a quantum walk with initial state $|{\psi }\rangle _0=|{0,R}\rangle /\sqrt{2}+i|{0,L}\rangle /\sqrt{2}$ ($R_{0,0}=1/\sqrt{2}$ and $L_{0,0}=i/\sqrt{2}$), and coin depending on $\Phi (t)=\frac{1}{4}\sin {(\omega t)}+\frac{1}{3}\sin {(2\omega t)}+\frac{1}{2}\sin {(3\omega t)}+\sin {(4\omega t)}$ over 300 steps. The graph on the left shows the result of the numerical simulation, and the graph on the right shows the result of the analytic solution with $w=0.4$.

So far we have discussed the dynamics of quantum walks with specific time-dependent coins and clarified the physical origin behind the quantum walk. Finally, we formulate the quantum walk for an arbitrary time-dependent coin, and discuss the controllability of the quantum walk. We employ the coin with *general* time dependence in a unitary form $\rho (t)=\cos ^2(\Phi (t))$ with

17 $$\begin{aligned} \Phi (t)&=\sum _{l=1}^{j}q_l\sin {(l\omega t)}, \end{aligned}$$

where $q_l$ is the Fourier coefficient. The time-dependent phase $\Phi (t)$ can express an arbitrary function. Through procedures parallel to those described above, we obtain the trajectory expressed as

18 $$\begin{aligned} x_c=\mp \int _{0}^{\tau }\cos {\Phi (\tau ')}d\tau '=Q_0+\sum _{K=-\infty (K\ne 0)}^{\infty }Q_K\sin {(K\omega \tau )} \end{aligned}$$

where

19 $$\begin{aligned} Q_0&=\sum _{k_2}\sum _{k_3}\cdot{\mkern -4mu}\cdot{\mkern -4mu}\cdot \sum _{k_j}\left( J_{-2k_2-3k_3\cdot{\mkern -4mu}\cdot{\mkern -4mu}\cdot -jk_j}(q_1)J_{k_2}(q_2)\cdot{\mkern -4mu}\cdot{\mkern -4mu}\cdot J_{k_j}(q_j)\tau \right) \end{aligned}$$

20 $$\begin{aligned} Q_K&= \sum _{K=-\infty (K\ne 0)}^{\infty }\sum _{k_2}\cdot{\mkern -4mu}\cdot{\mkern -4mu}\cdot \sum _{k_j}\frac{J_{K-2k_2-\cdot{\mkern -4mu}\cdot{\mkern -4mu}\cdot -jk_j}(q_1)J_{k_2}(q_2)\cdot{\mkern -4mu}\cdot{\mkern -4mu}\cdot J_{k_j}(q_j)}{K\omega } \end{aligned}$$

with $K=k_1+2k_2+\cdot{\mkern -4mu}\cdot{\mkern -4mu}\cdot +jk_j$. Eq. (18) is nothing but a Fourier series expansion and is the central result of this paper. The implication of Eq. (18) is that walker’s trajectory can take any path. Therefore, we can obtain the desired trajectory through designing the coin operator. This is another significant result of this paper. Figure 4 shows one such example with a coin depending on $\Phi (t)=\frac{1}{4}\sin {(\omega t)}+\frac{1}{3}\sin {(2\omega t)}+\frac{1}{2}\sin {(3\omega t)}+\sin {(4\omega t)}$. The the analytic solutions replicates the numerical simulations.

## Discussion

In summary, quantum walks with arbitrary time-dependent coins have been studied both numerically and analytically. The numerical simulations have been reproduced almost perfectly by the analytical solutions, and together have revealed the walking mechanism hidden in the quantum walks in the wave picture, i.e., that the coin flipping rate plays the role of the wave speed of the quantum walk governing the walker’s trajectories. This wave behavior can also be interpreted as Floquet oscillations in periodic energy bands in the particle picture. Based on this walking mechanism, the walker’s trajectory has been proved to be represented by the Fourier series of coins. Therefore, the walking mechanism enables us to tailor quantum walks as we desire through manipulating the coin flipping rate. Our results open the path towards the control of quantum walks.

## Methods

### Analytical solutions

In the supplement, we show our analytic solution for predicting the probability distribution of the walker’s location for quantum walks with a time-dependent coin. According to Bañuls’s extended theory^21^ which is based on the seminal work of Knight et al.^26^, the time evolution of the probability amplitudes is given by

21 $$\begin{aligned} R_{m,n+1}&=\cos \theta _n R_{m-1,n}+\sin \theta _n L_{m-1,n}, \end{aligned}$$

22 $$\begin{aligned} L_{m,n+1}&=\sin \theta _n R_{m+1,n}-\cos \theta _n L_{m+1,n}. \end{aligned}$$

We derive recurrence formulas of $R_{m,n}$ and $L_{m,n}$ by using Eqs. (21) and (22),

23 $$\begin{aligned} S_{n-1}R_{m,n+1}-S_n R_{m,n-1}&=C_n S_{n-1} R_{m-1,n}+S_n C_{n-1}R_{m+1,n}, \end{aligned}$$

24 $$\begin{aligned} S_{n-1}L_{m,n+1}-S_n L_{m,n-1}&=S_n C_{n-1} L_{m-1,n}+C_n S_{n-1}L_{m+1,n}, \end{aligned}$$

where $S_n\equiv \sin {\theta _n}$ and $C_n\equiv \cos {\theta _n}$. These (for $A_{m,n} = R_{m,n}$ or $L_{m,n}$) can be rewritten as

25 $$\begin{aligned}&S_n^{+}C_n^{-}(A_{m,n+1}-A_{m,n-1})-C_n^{+}S_n^{-}(A_{m,n+1}+A_{m,n-1})\nonumber \\&\quad =S_n^{+}C_n^{+} (A_{m-1,n}-A_{m+1,n})-S_n^{-}C_n^{-} (A_{m-1,n}+A_{m+1,n},) \end{aligned}$$

by using the following relations,

26 $$\begin{aligned} S_n&=S_n^{+}C_n^{-}+C_n^{+}S_n^{-}, \end{aligned}$$

27 $$\begin{aligned} S_{n-1}&=S_n^{+}C_n^{-}-C_n^{+}S_n^{-},\end{aligned}$$

28 $$\begin{aligned} C_{n}&=C_n^{+}C_n^{-}-S_n^{+}S_n^{-},\end{aligned}$$

29 $$\begin{aligned} C_{n-1}&=C_n^{+}C_n^{-}+S_n^{+}S_n^{-}, \end{aligned}$$

where $C_n^{\pm }=\cos \theta _n^{\pm }$ and $S_n^{\pm }=\sin \theta _n^{\pm }$ are denoted with $\theta _n=\theta _n^{+}+\theta _n^{-} \text{ and } \theta _{n-1}=\theta _n^{+}-\theta _n^{-}.$ Since trigonometric functions are approximated as $S_n^{-}\simeq 0$, $C_n^{-}\simeq 1$, $S_n^{+}\simeq \sin {\theta _n}$, and $C_n^{+}\simeq \cos {\theta _n}$ under small $\theta _n^{\pm }$, the difference equation, Eq. (6), in the main content is expressed as

30 $$\begin{aligned} A_{m,n+1}-A_{m,n-1}&=-\cos {\theta _n}(A_{m+1,n}-A_{m-1,n}). \end{aligned}$$

By introducing a continuum field *A*(*x*, *t*) instead of the discrete field $A_{m,n}$, Eq. (30) can also be expressed in the Taylor expansion series as

31 $$\begin{aligned} \sum _{i=0}\frac{2T^{2i+1}}{(2i+1)!}\frac{\partial ^{2i+1}}{\partial t^{2i+1}}A(x,t)&=-\cos {\theta (t)}\left\{ \sum _{i=0}\frac{2X^{2i+1}}{(2i+1)!}\frac{\partial ^{2i+1}}{\partial x^{2i+1}}A(x,t)\right\} , \end{aligned}$$

where $\cos {\theta (t)}$ is a continuum function that replaces $\cos {\theta _n}$ . Keeping only the first few, lowest order terms, this reduces to

32 $$\begin{aligned} T\frac{\partial }{\partial t} A(x,t)=-\cos {\theta (t)} \left( X\frac{\partial }{\partial x}+\frac{X^{3}}{3!} \frac{\partial ^{3}}{\partial x^{3}}\right) A(x,t). \end{aligned}$$

Since Eq. (32) describes a wave that propagates in only one direction (according to linear wave theory), an auxiliary field $A_{m,n}^{\pm }$ (in discrete systems) that travels in both directions was originally required to preserve the symmetry of the system. After introducing the dimensionless variables $\xi$ and $\tau$, this results in

33 $$\begin{aligned} \frac{\partial }{\partial \tau }A^{\pm }(\xi ,\tau )=\mp \cos {\theta (\tau )} \left( \frac{\partial }{\partial \xi }+\frac{1}{3!}\frac{\partial ^{3}}{\partial \xi ^{3}}\right) A^{\pm }(\xi ,\tau ). \end{aligned}$$

To obtain an analytical solution for the partial differential equation in Eq. (33), we adopt the usual method of solving partial differential equations using the Fourier method and assume $A^{\pm }(\xi ,\tau )$ with functions $G_m(\xi )$ and $A_m^{\pm }(\tau )$ in the variable-separated form,

34 $$\begin{aligned} A^{\pm }(\xi ,\tau )&=\sum _{m}G_m(\xi )A_m^{\pm }(\tau ), \end{aligned}$$

35 $$\begin{aligned} G_m(\xi )&=N\exp {\left[ -\frac{(\xi -m)^2}{4w^2}\right] }, \end{aligned}$$

with a normalization factor *N*. By using the Fourier transformation from the wavenumber *k* space,

36 $$\begin{aligned} A^{\pm }(\xi ,\tau )=\int _{-\infty }^{\infty }e^{ik\xi }A^{\pm }(k,\tau )dk , \end{aligned}$$

the partial differential equation, Eq. (33), reduces to the ordinary differential equation

37 $$\begin{aligned} \frac{\partial A^{\pm }(k,\tau )}{\partial \tau } =\mp \cos {\theta (\tau )}\left( ik-\frac{1}{6}ik^3\right) A^{\pm }(k,\tau ). \end{aligned}$$

Equation (37) can be immediately solved as

38 $$\begin{aligned} A^{\pm }(k,\tau )=A^{\pm }(k,0)e^{\mp ig(k,\tau )}, \end{aligned}$$

where

39 $$\begin{aligned} g(k,\tau )=\left( k-\frac{1}{6}k^3\right) s(\tau ). \end{aligned}$$

To find $A^{\pm }(k,0)$ in Eq. (38), we perform inverse Fourier transformations on Eq. (36) by setting $\tau =0$, which yields

40 $$\begin{aligned} A^{\pm }(k,0)=\int _{-\infty }^{\infty }e^{-ik\xi }A^{\pm }(\xi ,0)d\xi , \end{aligned}$$

where

41 $$\begin{aligned} A^{\pm }(\xi ,0)=\sum _{m}G_m(\xi )A_{m,0}^{\pm } \end{aligned}$$

with $A_m^{\pm }(0)=A_{m,0}^{\pm }$. Since the Gaussian integral is given as

42 $$\begin{aligned} \int _{-\infty }^{\infty }e^{-ik\xi }G_{m}(\xi )d\xi =N\sqrt{4w^2\pi }e^{-imk-k^2w^2}, \end{aligned}$$

Eq. (40) reduces to

43 $$\begin{aligned} A^{\pm }(k,0)&=e^{-k^2w^2}\sum _{m}A_{m,0}^{\pm }e^{-imk}, \end{aligned}$$

where the prefactor of the Gaussian integral $N\sqrt{4w^2\pi }$ is omitted in the following because it is merely a scaling factor. Substituting Eq. (43) into Eq. (38), $A^{\pm }(k,\tau )$ is rewritten as

44 $$\begin{aligned} A^{\pm }(k,\tau )&=e^{\mp ig(k,\tau )}e^{-k^2w^2}\sum _{m}A_{m,0}^{\pm }e^{-imk}. \end{aligned}$$

Therefore, $A^{\pm }(\xi ,\tau )$ in Eq. (36) can be expressed by

45 $$\begin{aligned} A^{\pm }(\xi ,\tau )&=\int _{-\infty }^{\infty }e^{-ik\xi }A^{\pm }(\xi ,\tau )d\xi \nonumber \\&=\sum _{m}A_{m,0}^{\pm }Z^{\pm }(\xi -m,\tau ) \end{aligned}$$

where

46 $$\begin{aligned} Z^{\pm }(\xi -m,\tau ) =\int _{-\infty }^{\infty }e^{ik(\xi -m)\mp ig(k,\tau )-k^2w^2}dk. \end{aligned}$$

$Z^{\pm }(\xi ',\tau )$ can be represented using existing analytical functions as follows

47 $$\begin{aligned} Z^{\pm }(\xi ',\tau )&=\int _{-\infty }^{\infty }e^{ik\xi ' \mp ig(k,\tau )-k^2w^2}dk\nonumber \\&=\int _{-\infty }^{\infty }e^{ik\left( \xi ' \mp s(\tau )\right) -k^2w^2\pm i\frac{k^3}{6}s(\tau )}dk, \end{aligned}$$

where $$\xi ' \equiv \xi -m$$.

Note that the relation $Z^{-}(\xi ',\tau )=Z^{+}(-\xi ',\tau )$ holds between $Z^{+}(\xi ',\tau )$ and $Z^{-}(\xi ',\tau )$. By using the Airy function ${{\text {Ai}}}(x)$,

48 $$\begin{aligned} {{\text {Ai}}}(x)=\int _{-\infty }^{\infty }e^{ikx+\frac{i}{3}k^3}dk. \end{aligned}$$

$Z^{+}(\xi ',\tau )$ is expressed as

49 $$\begin{aligned} Z^{\pm }(\xi ,\tau )=\left| \frac{2}{s(\tau )}\right| ^{\frac{1}{3}}e^{\chi }{{\text {Ai}}}(\zeta ), \end{aligned}$$

where

50 $$\begin{aligned} \zeta&=\left| \frac{2}{s(\tau )}\right| ^{\frac{1}{3}}\left( \pm \xi - s(\tau )+\frac{2w^4}{s(\tau )}\right) , \end{aligned}$$

51 $$\begin{aligned} \chi&=\frac{2 w^2}{s(\tau )}\left( \pm \xi - s(\tau )+\frac{4 w^4}{3s(\tau )}\right) , \end{aligned}$$

52 $$\begin{aligned} s(\tau )&=\mp \int _{0}^{\tau }\cos {\theta (\tau ')}d\tau '. \end{aligned}$$

Finally, let us consider the initial coefficients $A^{\pm }_{m,0}$ in Eq. (45). According to the relation $A_{m,n}\equiv A_{m,n} ^{+}+(-1)^m A_{m,n} ^{-}$ proposed by Knight et al., the following relations

$$\begin{aligned} A_{m,0}&= A_{m,0} ^{+}+ A_{m,0} ^{-}, \\ A_{m,1}&= A_{m,1} ^{+}- A_{m,1} ^{-}\simeq A_{m,0} ^{+}+ A_{m,0} ^{-}, \end{aligned}$$

lead to the simple relation

53 $$\begin{aligned} A_{m,0} ^{\pm }=\frac{1}{2}(A_{m,0}\pm A_{m,1}). \end{aligned}$$

Using this relation, we obtain

54 $$\begin{aligned} A^{\pm }(\xi ,\tau ) =\frac{1}{2}\sum _{m}(A_{m,0}\pm A_{m,1})Z^{\pm }(\xi -m,\tau ). \end{aligned}$$

Suppose that the walker starts at $m=0$, then the initial condition

$$\begin{aligned} {\left\{ \begin{array}{ll} A_{m,n}\ne 0, &{} (m,n)=(0,0),(\pm 1,1) \\ A_{m,n}= 0, &{} \text{ otherwise } \end{array}\right. } \end{aligned}$$

leads to

55 $$\begin{aligned} A_{-1,0} ^{\pm }&=\frac{1}{2}(A_{-1,0}\pm A_{-1,1}) =\frac{1}{2}A_{-1,1}, \end{aligned}$$

56 $$\begin{aligned} A_{0,0} ^{\pm }&=\frac{1}{2}(A_{0,0}\pm A_{0,1}) =\frac{1}{2}A_{0,0},\end{aligned}$$

57 $$\begin{aligned} A_{1,0} ^{\pm }&=\frac{1}{2}(A_{1,0}\pm A_{1,1}) =\pm \frac{1}{2}A_{1,1},\end{aligned}$$

58 $$\begin{aligned} A_{m,0}^{\pm }&=0\quad (n\ne -1, 0, 1). \end{aligned}$$

As a result, Eq. (8) reduces to

59 $$\begin{aligned} A^{\pm }(\xi ,\tau )&=\sum _{m=-1}^{1}A^{\pm }_{m,0}Z^{\pm }(\xi -m,\tau )\nonumber \\&=\frac{1}{2}\sum _{m=-1}^{1}(A_{m,0}\pm A_{m,1})Z^{\pm }(\xi -m,\tau ). \end{aligned}$$

Thus, we have obtained an analytic solution to quantum walks with a time-dependent coin that is essentially the same as the solution of Bañuls et al. except for the difference in the coin operators.

Based on the above elementary results, let us now derive the analytical solution for the quantum walks with the *generalized* time-dependent coins in a unitary form using $\rho (t)=\cos ^2(\Phi (t)))$ in the same manner as before. We start from the recurrence formulas Eq. (25) replacing $\theta _n$ with $\Phi _n(=\sum _{l=1}^{j}q_l\sin {(nl\omega T)})$. Since trigonometric functions are approximated as $S_n^{-}\simeq 0$, $C_n^{-}\simeq 1$, $S_n^{+}\simeq \sin {\Phi _n}$, and $C_n^{+}\simeq \cos {\Phi _n}$ under small $\Phi _n^{\pm }$, the difference equation is expressed as

60 $$\begin{aligned} A_{m,n+1}-A_{m,n-1}=\cos \Phi _n(A_{m-1,n}-A_{m+1,n}). \end{aligned}$$

Then we obtain the wave equation in the continuum limit,

61 $$\begin{aligned} \frac{\partial }{\partial \tau }A^{\pm }(\xi ,\tau )=\mp \cos {\Phi (\tau )}\left( \frac{\partial }{\partial \xi }+\frac{1}{3!}\frac{\partial ^{3}}{\partial \xi ^{3}}\right) A^{\pm }(\xi ,\tau ). \end{aligned}$$

Since the velocity of the wave corresponds to $\mp \cos {\Phi (\tau )}$ in the above equation, the walker’s trajectory is described by the integral of the velocity over a defined interval as follows,

62 $$\begin{aligned} x_c&= \int _{0}^{\tau }\cos {\Phi (\tau ')}d\tau '\nonumber \\&= \int _{0}^{\tau }\cos {\left( \sum _{l=1}^{j}q_l\sin {(l\omega \tau ')}\right) }d\tau '. \end{aligned}$$

Before proceeding with the integration, let us prove the following relations by using mathematical induction,

63 $$\begin{aligned} \cos {\left( \sum _{l=1}^{j}q_l\sin {(l\omega t)}\right) }&=\sum _{k_1,k_2,\cdot{\mkern -4mu}\cdot{\mkern -4mu}\cdot ,k_j}J_{k_1}(q_1)J_{k_2}(q_2)\cdot{\mkern -4mu}\cdot{\mkern -4mu}\cdot J_{k_j}(q_j)\cos \{\omega t(k_1+2k_2+\cdot{\mkern -4mu}\cdot{\mkern -4mu}\cdot +jk_j)\}, \end{aligned}$$

64 $$\begin{aligned} \sin {\left( \sum _{l=1}^{j}q_l\sin {(l\omega t)}\right) }&=\sum _{k_1,k_2,\cdot{\mkern -4mu}\cdot{\mkern -4mu}\cdot , k_j}J_{k_1}(q_1)J_{k_2}(q_2)\cdot{\mkern -4mu}\cdot{\mkern -4mu}\cdot J_{k_j}(q_j)\sin \{\omega t(k_1+2k_2+\cdot{\mkern -4mu}\cdot{\mkern -4mu}\cdot +jk_j)\} , \end{aligned}$$

where $J_{k_l}(q_l)$ is the Bessel function of the first kind of order $k_l$ of $q_l$. First, at $j=1$, the equations are

65 $$\begin{aligned} \cos {\left( q_1\sin {(\omega t)}\right) }&=\mathfrak {R}{\left( \exp [iq_1\sin {(\omega t)}]\right) } =\sum _{k_1}J_{k_1}(q_1)\cos (k_1\omega t), \end{aligned}$$

66 $$\begin{aligned} \sin {\left( q_1\sin {(\omega t)}\right) }&=\mathfrak {I}{\left( \exp [iq_1\sin {(\omega t)}]\right) } =\sum _{k_1}J_{k_1}(q_1)\sin (k_1\omega t). \end{aligned}$$

Thus, Eqs. (63) and (64) are established with certainty. Assuming that Eqs. (63) and (64) are correct at $j=p$, we show that the relations also hold at $j=p+1$. At $j=p+1$ we obtain the relations for Eq. (63) as follows,

67 $$\begin{aligned}{}&\cos {\left\{ \sum _{l=1}^{p+1}q_l\sin {(l\omega t)}\right\} }\nonumber \\&\quad = \cos {\left\{ \sum _{l=1}^{p}q_l\sin {(l\omega t)}\right\} }\cos {\left\{ q_{p+1}\sin {((p+1)\omega t)}\right\} } \nonumber \\&\qquad -\sin {\left\{ \sum _{l=1}^{p}q_l\sin {(l\omega t)}\right\} }\sin {\left\{ q_{p+1}\sin {((p+1)\omega t)}\right\} }\nonumber \\&\quad = \sum _{k_1,\cdot{\mkern -4mu}\cdot{\mkern -4mu}\cdot , k_p}J_{k_1}(q_1)\cdot{\mkern -4mu}\cdot{\mkern -4mu}\cdot J_{k_p}(c_p)\cos \{\omega t(k_1+\cdot{\mkern -4mu}\cdot{\mkern -4mu}\cdot +pk_p)\}\sum _{k_{p+1}}J_{k_{p+1}}(q_{p+1})\cos {(\omega t(p+1)k_{p+1})}\nonumber \\&\qquad -\sum _{k_1,\cdot{\mkern -4mu}\cdot{\mkern -4mu}\cdot , k_p}J_{k_1}(q_1)\cdot{\mkern -4mu}\cdot{\mkern -4mu}\cdot J_{k_p}(c_p)\sin \{\omega t(k_1+\cdot{\mkern -4mu}\cdot{\mkern -4mu}\cdot +pk_p)\}\sum _{k_{p+1}} J_{k_{p+1}}(q_{p+1})\sin {(\omega t(p+1)k_{p+1})}\nonumber \\&\quad = \sum _{k_1,\cdot{\mkern -4mu}\cdot{\mkern -4mu}\cdot , k_{p+1}}J_{k_1}(q_1)\cdot{\mkern -4mu}\cdot{\mkern -4mu}\cdot J_{k_{p+1}}(q_{p+1})\left[ \cos \{\omega t(k_1+\cdot{\mkern -4mu}\cdot{\mkern -4mu}\cdot +pk_p)\} \cos {(\omega t(p+1)k_{p+1})} \right. \nonumber \\&\qquad \quad \left. -\sin \{\omega t(k_1+\cdot{\mkern -4mu}\cdot{\mkern -4mu}\cdot +pk_p)\} \sin {(\omega t(p+1)k_{p+1})}\right] \nonumber \\&\quad = \sum _{k_1,\cdot{\mkern -4mu}\cdot{\mkern -4mu}\cdot , k_{p+1}}J_{k_1}(q_1)\cdot{\mkern -4mu}\cdot{\mkern -4mu}\cdot J_{k_{p+1}}(q_{p+1})\cos \{\omega t(k_1+\cdot{\mkern -4mu}\cdot{\mkern -4mu}\cdot +pk_p+(p+1)k_{p+1})\}, \end{aligned}$$

demonstrating that Eq. (63) is true. Therefore, we confirm that Eq. (63) is established for any natural number *p*. In the same way, it can be shown that Eq. (64) is also correct. The trajectory Eq. (62) is rewritten as

68 $$\begin{aligned} x_c&= \int _{0}^{\tau }\sum _{k_1,k_2,\cdot{\mkern -4mu}\cdot{\mkern -4mu}\cdot ,k_j}J_{k_1}(q_1)J_{k_2}(q_2)\cdot{\mkern -4mu}\cdot{\mkern -4mu}\cdot J_{k_j}(q_j)\cos \{\omega \tau '(k_1+2k_2+\cdot{\mkern -4mu}\cdot{\mkern -4mu}\cdot +jk_j)\} d\tau '\nonumber \\&= \int _{0}^{\tau }\sum _{k_1,\cdot{\mkern -4mu}\cdot{\mkern -4mu}\cdot ,k_j}J_{k_1}(q_1)\cdot{\mkern -4mu}\cdot{\mkern -4mu}\cdot J_{k_j}(q_j)\cos (K\omega \tau ') d\tau '\nonumber \\&= \sum _{k_1,\cdot{\mkern -4mu}\cdot{\mkern -4mu}\cdot ,k_j(K\ne 0)}J_{k_1}(q_1)\cdot{\mkern -4mu}\cdot{\mkern -4mu}\cdot J_{k_j}(q_j)\left[ \frac{1}{K\omega }\sin (K\omega \tau ')\right] _{0}^{\tau }+\sum _{k_1,\cdot{\mkern -4mu}\cdot{\mkern -4mu}\cdot ,k_j(K=0)}J_{k_1}(q_1)\cdot{\mkern -4mu}\cdot{\mkern -4mu}\cdot J_{k_j}(q_j)\tau \nonumber \\&= \sum _{k_1,\cdot{\mkern -4mu}\cdot{\mkern -4mu}\cdot ,k_j(K\ne 0)}\frac{J_{k_1}(q_1)\cdot{\mkern -4mu}\cdot{\mkern -4mu}\cdot J_{k_j}(q_j)}{K\omega }\sin (K\omega \tau )+\sum _{k_1,\cdot{\mkern -4mu}\cdot{\mkern -4mu}\cdot ,k_j(K=0)}J_{k_1}(q_1)\cdot{\mkern -4mu}\cdot{\mkern -4mu}\cdot J_{k_j}(q_j),\tau \end{aligned}$$

with $K=k_1+2k_2+\cdot{\mkern -4mu}\cdot{\mkern -4mu}\cdot +jk_j$. By using the relation

69 $$\begin{aligned} \prod _{i=1}^{j}\left( \sum _{k_i=-\infty }^\infty J_{k_i}(c_i)\right)&=\sum _{K=-\infty }^{\infty }\sum _{k_2}\cdot{\mkern -4mu}\cdot{\mkern -4mu}\cdot \sum _{k_j}J_{K-2k_2-\cdot{\mkern -4mu}\cdot{\mkern -4mu}\cdot -jk_j}(q_1)J_{k_2}(q_2)\cdot{\mkern -4mu}\cdot{\mkern -4mu}\cdot J_{k_j}(q_j), \end{aligned}$$

the walker’s trajectory of quantum walks with *generalized* time-dependent coin is expressed as

70 $$\begin{aligned} x_c&=Q_0+\sum _{K=-\infty (K\ne 0)}^{\infty }Q_K\sin {(K\omega \tau )}, \end{aligned}$$

where

71 $$\begin{aligned} Q_0&=\sum _{k_2}\sum _{k_3}\cdot{\mkern -4mu}\cdot{\mkern -4mu}\cdot \sum _{k_j}\left( J_{-2k_2-3k_3\cdot{\mkern -4mu}\cdot{\mkern -4mu}\cdot -jk_j}(q_1)J_{k_2}(q_2)\cdot{\mkern -4mu}\cdot{\mkern -4mu}\cdot J_{k_j}(q_j)\tau \right) , \end{aligned}$$

72 $$\begin{aligned} Q_K&= \sum _{K=-\infty (K\ne 0)}^{\infty }\sum _{k_2}\cdot{\mkern -4mu}\cdot{\mkern -4mu}\cdot \sum _{k_j}\frac{J_{K-2k_2-\cdot{\mkern -4mu}\cdot{\mkern -4mu}\cdot -jk_j}(q_1)J_{k_2}(q_2)\cdot{\mkern -4mu}\cdot{\mkern -4mu}\cdot J_{k_j}(q_j)}{K\omega }. \end{aligned}$$

This is one of the central results of this paper. Thus, we can control, in general manner, the walker’s trajectory in quantum walks even with time-dependent coins.

## Data Availability

The data that support the findings of this study are available from the corresponding author upon request.
