# Publisher Correction: Floquet-engineered quantum walks

**DOI:** 10.1038/s41598-021-85564-0

**Published:** 2021-03-17

**Authors:** Haruna Katayama, Noriyuki Hatakenaka, Toshiyuki Fujii

**Affiliations:** 1 Graduate School of Integraged Arts and Sciences, Hiroshima University, Higashihiroshima, 739-8521 Japan; 2 Department of Physics, Asahikawa Medical University, Midorigaoka-higashi, Asahikawa, 078-8510 Japan

Correction to: *Scientific Reports* 10.1038/s41598-020-74418-w, published online 16 October 2020

This Article contains an error in Figure 2 where Greek letters are replaced with Helvetica font. The correct Figure 2 appears below as Figure 1.

**Figure 1:**
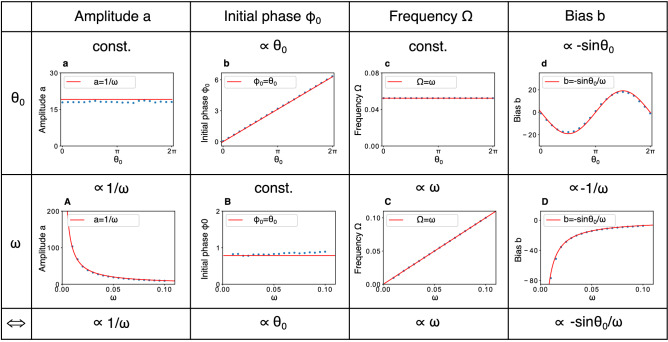
A correct version of the original Figure 2.

